# Chromatin assembly from nucleosome to heterochromatin: the issue of DNA damage

**DOI:** 10.1186/1756-8935-6-S1-O15

**Published:** 2013-03-18

**Authors:** Geneviève Almouzni

**Affiliations:** 1 Laboratory of Nuclear Dynamics and Genome Plasticity, UMR 218 CNRS/ Institut Curie, Research Center, 26 rue d’Ulm, F-75248 Paris cedex 05, France

## 

Studies concerning the mechanism of DNA replication and repair have advanced our understanding of the stable transmission through multiple cell cycles of a faithful genetic material. Recent work has shed light on possible means to ensure the stable transmission of information beyond just DNA and the concept of epigenetic inheritance has emerged. Considering chromatin-based information, key candidates have arisen as epigenetic marks including DNA and histone modifications, histone variants, non-histone chromatin proteins, nuclear RNA as well as higher-order chromatin organization. Thus, understanding the dynamics and stability of these marks following disruptive events during replication and repair and throughout the cell cycle becomes of critical importance for the maintenance of any given chromatin state.

We will present our most recent work on this topic, considering mostly histone variant dynamics during the cell-cycle and in response to DNA damage.
